# Validation of Reference Genes for Relative Quantitative Gene Expression Studies in Cassava (*Manihot esculenta* Crantz) by Using Quantitative Real-Time PCR

**DOI:** 10.3389/fpls.2016.00680

**Published:** 2016-05-19

**Authors:** Meizhen Hu, Wenbin Hu, Zhiqiang Xia, Xincheng Zhou, Wenquan Wang

**Affiliations:** ^1^College of Agriculture, Hainan UniversityHaikou, China; ^2^The Institute of Tropical Bioscience and Biotechnology, Chinese Academy of Tropical Agricultural SciencesHaikou, China; ^3^Key Laboratory of Biology and Genetic Resources of Tropical Crops, Ministry of AgricultureHaikou, China; ^4^Tropical Crops Genetic Resources Institute, Chinese Academy of Tropical Agricultural SciencesDanzhou, China

**Keywords:** cassava, reference gene, quantitative real-time PCR, geNorm, NormFinder, drought stress

## Abstract

Reverse transcription quantitative real-time polymerase chain reaction (real-time PCR, also referred to as quantitative RT-PCR or RT-qPCR) is a highly sensitive and high-throughput method used to study gene expression. Despite the numerous advantages of RT-qPCR, its accuracy is strongly influenced by the stability of internal reference genes used for normalizations. To date, few studies on the identification of reference genes have been performed on cassava (*Manihot esculenta* Crantz). Therefore, we selected 26 candidate reference genes mainly via the three following channels: reference genes used in previous studies on cassava, the orthologs of the most stable *Arabidopsis* genes, and the sequences obtained from 32 cassava transcriptome sequence data. Then, we employed ABI 7900 HT and SYBR Green PCR mix to assess the expression of these genes in 21 materials obtained from various cassava samples under different developmental and environmental conditions. The stability of gene expression was analyzed using two statistical algorithms, namely geNorm and NormFinder. geNorm software suggests the combination of *cassava4.1_017977* and *cassava4.1_006391* as sufficient reference genes for major cassava samples, the union of *cassava4.1_014335* and *cassava4.1_006884* as best choice for drought stressed samples, and the association of *cassava4.1_012496* and *cassava4.1_006391* as optimal choice for normally grown samples. NormFinder software recommends *cassava4.1_006884* or *cassava4.1_006776* as superior reference for qPCR analysis of different materials and organs of drought stressed or normally grown cassava, respectively. Results provide an important resource for cassava reference genes under specific conditions. The limitations of these findings were also discussed. Furthermore, we suggested some strategies that may be used to select candidate reference genes.

## Introduction

Analysis of mRNA expression patterns is one of the most commonly used molecular techniques to verify the function of a candidate gene. Real-time PCR (also referred to as quantitative RT-PCR) is an extremely sensitive and cost–effective method employed to study gene expression changes (Gachon et al., 2004). qPCR achieves absolute quantification and relative quantification. Assays based on linear regression of efficiency (LRE; Rutledge and Stewart, 2010; Rutledge, 2011) and droplet digital PCR (Vogelstein and Kinzler, 1999; Pinheiro et al., 2012) allow the accurate quantification of a target gene at a single molecule sensitivity. Relative qPCR is a useful technology to compare gene expression levels, although this method suffers from several systematic errors, such as DNA contamination, and to select stably expressed reference genes (Udvardi et al., 2008). Stable reference genes (also called housekeeping genes) can normalize and control many variables, such as different amounts and qualities of starting materials or the differences among tissues or samples treated under various conditions.

During the pre-genomic era, researchers selected some genes serving as internal controls in basic cellular processes, including 18S RNA, glyceraldehyde-3-phosphate dehydrogenase, beta-actin genes, and tubulin (Goidin et al., 2001; Kim et al., 2003). However, numerous studies reported that the transcript levels of traditional reference genes can vary considerably under different experimental conditions (Thellin et al., 1999; Lee et al., 2002; Radonic et al., 2004; Czechowski et al., 2005). Until now, a number of strategies are used to select and validate reference genes. The expressed sequence tag (EST) database, transcriptome data and whole-genome GeneChip data comprise a large set of data sources used to select candidate reference genes. In tomato, several classical reference genes were proposed as internal controls based on the relative abundance of their EST database (Coker and Davies, 2003). However, compared with novel reference genes, three of the abovementioned classical reference genes displayed unstable expression in a set of 27 tomato samples (Exposito-Rodriguez et al., 2008). In barley, several novel candidate reference genes were selected though a combined strategy of “*in silico*” transcriptome analysis combined with web search optimization (Faccioli et al., 2007). Novel superior internal controls were identified via genome-wide screening, and most of them were expressed at much lower levels than traditional reference genes (Czechowski et al., 2005). Based on the work of Czechowski et al. (2005), these superior reference genes have been successfully employed to search for orthologs in unrelated species, such as tomato (Exposito-Rodriguez et al., 2008; Dekkers et al., 2012), *Eucalyptus* (Cassan-Wang et al., 2012), and desert moss *Syntrichia caninervis* (Li et al., 2015).

Most reference gene evaluation studies of plants have been performed on model species. However, few works have focused on cassava. Cassava (*Manihot esculenta* Crantz) is a crop thriving in tropical and subtropical regions because of its starchy tuberous root, which is edible and a major source of carbohydrates. A study has evaluated the stable reference genes to quantitate potyvirus in cassava (Moreno et al., 2011). However, the research only focused on CBSV-infected cassava, and only five genes were tested. Nevertheless, no genes were identified with stable expression in cassava across a wide range of developmental stages and varied conditions.

In the present work, we identified some novel candidate reference genes in cassava; these genes can be suitably used to normalize gene expression levels at different development phases and under drought stress. Three genes, namely, *tubulin* (*TUB*; Yao et al., 2014), *protein phosphatase 2A* (*PP2A*), and *UBQ10* (Moreno et al., 2011) were selected through bibliographic reviews of studies on gene expression in cassava. Six genes that are putative orthologs of the top six reference genes in *Arabidopsis*, as defined by Czechowski et al. (2005), were also selected. In addition an *ACT* gene was included. Moreover, we took full advantage of the 32 transcriptional databases created in our laboratory and divided these into three series. The selection method followed the rule described by Czechowski et al. (2005). In each series, the mean expression value (MV), SD, and the radio SD/MV ratio (coefficient of variation, CV) of each gene were subsequently calculated and sorted according to their CV value. A smaller CV value of a gene expression profile indicates a more stable expression. After comparing the varying CV values in the three series, 16 candidate genes were selected. The expression of the 26 genes was assessed by qRT-PCR from 21 tissues and materials of cassava plants that were normally grown or subjected under drought stress. The stability of gene expression was estimated using two statistical approaches, namely, geNorm (Vandesompele et al., 2002), and NormFinder (Andersen et al., 2004).

## Materials and Methods

### Candidate Gene Selection

We first selected three potential reference genes, namely, *TUB*, *PP2A*, and *UBQ10*, from previous studies on cassava gene expression profiles. An *ACT* gene was added as a candidate gene. On the basis of previous reports on the model plant *Arabidopsis*, we surveyed the cassava Phytozome database for putative orthologs of the top six reference genes (Czechowski et al., 2005). The gene IDs of the top six reference genes in *Arabidopsis* were *AT2G28390*, *AT4G34270*, *AT4G26410*, *AT4G33380*, *AT5G46630*, and *AT5G55840*.

We used our 32 transcriptional data to identify the candidate reference genes in cassava grown under a broad range of developmental and environmental conditions. The 32 transcriptional data contained 4 varieties (W14, KU50, Arg7, and SC124), covering 3 organs (leaf, tuberous, and stem), and including 11 drought stress conditions. The data were divided into three series containing developmental series (a total of 10 transcriptome data from W14, KU50, and Arg7), Arg7 drought stress series (a total of five controls and five treated materials), and SC124 drought series (a total of six controls and six treated materials). We used the method described by Czechowski et al. (2005); MVs, SDs, and CVs were calculated for each gene in each series. The genes were sorted according to their CV value in ascending order, and 100 genes showing the lowest CV value in each experimental series were selected. By analyzing the CV value and the annotation of the selected 100 genes in each series, we chose 16 candidate genes showing the lowest CV values in all three series. To analyze the result of the top 100 invariant genes in the whole transcriptomic dataset vs. the invariant genes in three subsets, the top 100 stably expressed genes were ranked by the same method. Supplementary Table S1 shows the description of the samples of transcriptome data. And the 32 transcriptome data are presented in Supplementary Excel 1. Supplementary Excel 1 also presents the top 100 genes, the traditional reference genes, and six orthologs of the top six stably expressed genes in *Arabidopsis* displaying the minimum CV value in the 3 subsets and of a total of 32 data.

### RT-PCR Primer Design and Test

Primers for *TUB*, *PP2A*, and *UBQ10* were adopted from literature. For other genes, gene models and information were downloaded from Phytozome^1^. The qPCR primer for these sequences was designed using^2^ Primer3 and according with the reported criteria (Czechowski et al., 2005). The specificity of the resulting primer pair sequences was checked against the cassava (*M. esculenta* Crantz) transcript database^1^ by using Blast (2.2.16+). Primer specificity was assessed by melting-curve analysis after RT-PCR and gel electrophoresis analyses of the amplicons.

### Plant Materials and Treatment

Cassava plants were grown in the experimental base of the Institute of Tropical Biosciences and Biotechnology in Haikou, China. All 21 tested samples were divided into three series (Supplementary Table S2): 12 normally grown samples, six drought stressed samples, and three samples that suffered from disease or nitrogen deficiency. Three planting patterns were adopted: field planting, planting in plots, and solution culture. Solution cultured plants were treated with 0.5x Afdaling nutrient with or without NH_4_NO_3_ under continuous oxygen aeration. The Afdaling nutrient solution was changed once every 2 days. We adopted 3 × 4 combination for material preparation and collection, three independent experimental trials, and four plants (four biological replicates) for each trial. Samples were harvested from each plant, immediately frozen in liquid nitrogen, and then stored at -80°C prior to analysis. The samples obtained from each plant were ground independently. Approximately 100 mg of the pulverized sample of the four biological repeat plants was subsequently mixed and used as an experimental sample. So we obtained 400 mg of mixture powder for each independent trail. To reduce the cost when validating reference gene, we mixed the powder from the three independent trails and finally obtained 1200 mg of mixed powder, which was used as sample for RNA extraction and validation of the reference genes. To test the target genes, we independently used the powder of three independent trials for RNA extraction and qRT-PCR.

Sufficient cassava plants were planted in soil with a row spacing of 70 cm and individual spacing of 45 cm. Leaf, petiole and root samples were obtained. The second to fifth fully expanded leaves bellow the shoot were considered mature leaves. Twelve tissue samples/organs from normally grown plants were collected at the given time: young leaves of KU50 (10 weeks); fibrous roots of KU50 (10 weeks); tuberous roots of KU50 (4 months); mature leaves of KU50 (6 months); petiole of KU50 (6 months); mature leaves of KU50 (8 months); tuberous roots of KU50 (8 months); tuberous roots of KU50 (9.5 months); tuberous roots from Rongyong9 (3 months); tuberous roots from SC124 (6 months); tuberous roots from SC5 (6 months); and flowers from Arg7.

Six drought-stressed samples were collected. One sample was treated with PEG6000 and the other five were treated by withholding water when the plants had grown for 5 months in 35 cm × 40 cm × 45 cm plots. In PEG6000 treatment, the KU50 plants were grown in aquaponics and supplied with 0.5x Afdaling nutrient solution for 6 weeks, and then treated with 10% PEG6000 for 24 h. Finally, all fully expanded leaves from each plant were collected. For the five other drought treatments, the plants were grown in 35 cm × 40 cm × 45 cm plots for 5 months prior to the water-withholding treatment. Plants was suffered from drought stress by withholding water for consecutive days (3, 6, and 7 days). Mature leaves and stems were collected as samples. These samples were leaves of W14 drought treated for 3 days, leaves of Arg7 drought treated for 3 days, leaves of KU50 drought treated for 6 days, leaves of Arg7 drought treated for 7 days, and stems of SC124 drought treated for 4 days.

Three special samples were determined: leaves of KU50 (6 months old) infected with *cassava brown leaf spot*; leaves of KU50 (3 months old) infected with *Mononychellus mcgregori*; leaves from naturally grown plants suffering from nitrogen deficiency (grown in aquaponics supplied with 0.5x Afdaling nutrient solution for 6 weeks and then with the solution bwithout NH_4_NO_3_ for 2 weeks).

### RNA Extraction and cDNA Synthesis and Quality Control

The total RNA from all the tissue samples was isolated using RNAprep Pure Kit (code no. DP441; Tiangen, Beijing, China) accordance with manufacturer’s instructions. An additional step using chloroform–isoamyl alcohol (24:1) was performed to remove the protein contaminants. In the kit, the total RNA and DNA were bound to the membrane, and the DNA was digested by DNase I. Subsequently, the DNA fragments were washed away, and the total RNA was purified. RNA concentration and quality were measured using NanoDrop. Only high-quality samples (260/280 ratios of 1.9 to 2.1) were used for the analysis. The integrity of the RNA samples was determined on a 2% agarose gel. Moreover, every RNA sample was tested via PCR by using the primers (5′-CAGTGGTGGTTCCACTATGTTCC-3′ and 5′-CAAAATGATTGCGAGGAAAGTAA-3′) designed to amplify a 482-bp genomic fragment of an *ACT* gene (*cassava4.1_009807*). Samples with no PCR production of the genomic contamination were further analyzed.

PrimeScript^TM^ RT reagent Kit with gDNA Eraser (Perfect Real Time; TAKARA, Dalian, China) was used in cDNA synthesis. The first strand of cDNA was synthesized with 600 ng of total RNA in a final reaction volume of 20 μl, following the manufacturer’s instructions.

### qRT-PCR Conditions and Analysis

cDNA samples were diluted 20 times for qRT-PCR analysis. PCR reactions were performed in an optical 384-well plate equipped with an ABI PRISM 7900 HT sequence detection system (Applied Biosystems) by using SYBR Green to monitor dsDNA synthesis, as previously described (Czechowski et al., 2005). The reaction mixture contained 4 μl of 2x *Power* SYBR Green PCR Master Mix reagent (Applied Biosystems), 2 μl of diluted cDNA, and 200 nM of each gene-specific primer in a final volume of 8 μl. The standard thermal profile of the manual was used for all PCR reactions. Amplicon dissociation curves, i.e., melting curves, were recorded. Data were analyzed using SDS 2.4 software (Applied Biosystems). PCR efficiency (*E*) was evaluated using a standard curve generated via PCR by using a fivefold dilution series.

### Analysis of Gene Stability

To rank the stability of the tested genes, we used geNorm v.3.5 (Vandesompele et al., 2002) and NormFinder (Andersen et al., 2004). The geNorm statistical algorithm is based on pairwise variation of a single reference candidate gene relative to all other investigated genes (Vandesompele et al., 2002). Pairwise variation *V*_n_/*V*_n+1_ between two sequential normalization factors was calculated to determine the optimal number of reference genes. NormFinder evaluates the stability of the reference genes based on the expression variations of the candidate reference genes (Andersen et al., 2004). A third one, BestKeeper is an Excel-based tool using pairwise correlations for determination of stable reference genes, and frequently used (Pfaﬄ et al., 2004; Barsalobres-Cavallari et al., 2009; Demidenko et al., 2011; Barros Rodrigues et al., 2014). However, BestKeeper can only compare the expression level up to 10 candidate reference genes together with 10 target genes (Pfaﬄ et al., 2004). Therefore BestKeeper was excluded from this study.

### Determination of *UGT85K4/UTG85K5* and *Cassava4.1_004044* Expression Profiles

To investigate the effect of the choice of selected reference genes on normalization of target genes, two genes of interest were used as examples: *UGT85K4/UGT85K5* and *cassava4.1_004044*. *UGT85K4* and *UGT85K5* are paralogs encoding uridine diphosphate glycosyltransferase (UGT), displaying 96% amino acid identity (Kannangara et al., 2011). Given the high similarity of these two paralogs in gene sequences, only one primer pair was designed for RT-qPCR. UGT85K4 and UTG85K5 orthologs catalyzed the last enzymatic step in the biosynthetic pathway for the cyanogenic glucosides linamarin and lotaustralin in cassava (Jorgensen et al., 2005; Kannangara et al., 2011). The cyanoglucoside content in cassava is genetically controlled, and cassava may be classified as low, medium and high CN varieties (Nambisan, 2011). *UGT85K4* and *UGT85K5* are likely to be alternatively expressed in cassava varieties. *Cassava4.1_004044* encodes a putative member of the cassava *Nitrate transporters NRT1* according to the result of homologous alignment. In *Arabidopsis*, the nitrate transporter AtNRT1.1 contributes to drought susceptibility (Guo et al., 2003). In *Gossypium herbaceum*, nitrate transporter is likely to be involved in drought stress signaling and adaption (Ranjan et al., 2012). One *NRT1* gene in cassava, *cassava4.1_004044* displayed a relatively high expression in normally grown cassava. Thus we chose this gene as target to investigate nitrate transporter genes that are possibly involved in drought stress signaling and adaptation. The relative expression profiles were analyzed using the 2^-ΔΔCT^ method (Pfaﬄ, 2001). The expression levels in three biological replicates of three technical repeats per sample were measured. The primer pair for *UGT85K4/UGT85K5* was F_CCAATGATCTGCTGGCCTTTC and R-CAACACTGATGGCTTCTTCGG. Primer pair for *cassava4.1_004044* was F-CCAGAACAAAAGACCACCCTG and R-AAAAGTTCAGGCCACCCATTC.

## Results

### Selection of Candidate Reference Genes

The expression levels of 26 candidate reference genes were evaluated. Three genes, namely, *TUB*, *PP2A*, and *UBQ10*, are commonly considered stable reference genes and were used in previous studies on cassava. In this research, one *ACT* gene was added. We searched for the orthologs of the genes listed as stably expressed in a genome-wide investigation of *Arabidopsis* in cassava genome. These genes are the orthologs of *AT2G28390* (SAND family, *SAND*), *AT4G34270* (Tip41-like, *Tip41*), *AT4G26410* (unknown function, *Expressed2*), *AT4G33380* (unknown function, *Expressed1*), *AT5G46630* (clathrin adaptor complex submit, *CACS*), and *AT5G55840* (pentatricopeptide repeat superfamily protein, *PPR repeat*). Comparison of the CV values was performed to determine the top 100 stably expressed genes from different experimental series; we selected 16 candidates for the comparison. **Table 1** shows the information on the 26 candidates, including the gene ID of cassava 4.1 generation, *Arabidopsis thaliana* ortholog locus, and annotation.

**Table 1:** Description of cassava candidate reference genes.

Gene ID	*Arabidopsis thaliana* ortholog locus	*A. thaliana* annotation
cassava4.1_007598	AT5G12250.1	*TUB*, beta_6 tubulin
cassava4.1_012251	AT3G58500.1	*PP2A*, protein phosphatase 2A-4
cassava4.1_009650	AT4G05320.2	*UBQ10*, polyubiquitin 10
cassava4.1_009780	AT5G09810.1	*ATC*, actin 7
cassava4.1_003909	AT2G28390.1	SAND family protein
cassava4.1_030171	AT4G34270.1	TIP41-like family protein
cassava4.1_015407	AT4G26410.1	Uncharacterized conserved protein UCP022280
cassava4.1_011861	AT4G33380.1	Expressed
cassava4.1_010439	AT5G46630.2	Clathrin adaptor complexes medium subunit family protein, AP-2 complex subunit mu-1
cassava4.1_024813	AT5G55840.1	Pentatricopeptide repeat (PPR) superfamily protein
cassava4.1_003449	AT3G13772.1	Endosomal membrane proteins, EMP70
cassava4.1_006445	AT1G20200.1	PAM domain (PCI/PINT associated module) protein
cassava4.1_006884	AT4G38630.1	26S proteasome regulatory complex, subunit N10
cassava4.1_006391	AT5G63890.1	Histidinol dehydrogenase
cassava4.1_010236	AT4G27780.1	Acyl-CoA-binding protein
cassava4.1_012496	AT1G10430.1	Serine/threonine-PP2A catalytic subunit
cassava4.1_014646	AT1G20270.1	2-oxoglutarate (2OG) and Fe(II)-dependent oxygenase superfamily protein
cassava4.1_003988	AT1G43690.1	Uncharacterized conserved protein
cassava4.1_010665	AT4G33890.1	Expressed
cassava4.1_014335	AT3G62870.1	Ribosomal protein L7Ae/L30e/S12e/Gadd45 family protein
cassava4.1_014799	AT2G05840.1	20S proteasome subunit PAA2
cassava4.1_006776	AT3G48440.1	Zinc finger C-x8-C-x5-C-x3-H type family protein
cassava4.1_017977	AT1G17880.1	Nascent polypeptide-associated complex subunit beta****
cassava4.1_002641	AT5G66810.1	PTHR12864//PTHR12864:SF8 – RAN BINDING PROTEIN 9-RELATED // SUBFAMILY NOT NAMED
cassava4.1_004051	AT1G07540.1	TRF-like 2
cassava4.1_001927	AT2G47330.1	ATP-dependent RNA helicase DDX42

### Verification of Primer Specificity and Efficiency

**Table 2** shows the RT-qPCR primer sequences and amplicon characteristic of the 26 candidate reference genes. The sequence length ranged from 62 to 180 bp. The average amplification efficiency varied from 82.1 to 110%. The specificity of the PCR primers was tested using melting-curve analysis (Supplementary Figure S1), following RT-qPCR and gel electrophoresis analyses of the amplicons. A more stringent test for the specificity of PCR was performed by sequencing the product of two constitutive genes (*cassava4.1_001927* and *cassava4.1_017977*) and product of *UBQ10* primers tested in a previous study (Moreno et al., 2011; data not shown). In the three cases, the sequence of the PCR product matched that of the intended target cDNA, confirming the PCR specificity of the primer pairs.

**Table 2:** Primers and amplicons for each of the 26 genes.

Gene ID	Forward primer sequence [5′-3′]	Reverse primer sequence [5′-3′]	Amplicon length (bp)	Efficiency (%)
cassava4.1_007598	GTGGAGGAACTGGTTCTGGA	TGCACTCATCTGCATTCTCC	180	90.3
cassava4.1_012251	TGCAAGGCTCACACTTTCATC	CTGAGCGTAAAGCAGGGAAG	150	94.5
cassava4.1_009650	TGCATCTCGTTCTCCGATTG	GCGAAGATCAGTCGTTGTTGG	166	82.1
cassava4.1_009780	TTCGTGTCAAGGTGTCGTGA	GCCCTCTCATTTGCTGCAAT	149	107.7
cassava4.1_003909	GGGAAATGGGCCTCACAAAAC	GCAAGTGGATCAAATGCTGCA	105	108.3
cassava4.1_030171	AAGGGACACTCGCATGCATT	CTCTCCAGCAGCTTTCACGA	74	100.2
cassava4.1_015407	CCACAAGCAATTTCCCAGTTTAAAG	TGCACTCGTCAACTCCTCTTTA	75	108.1
cassava4.1_011861	GAACGCCAACATCACTGAGC	CATCCTGGCTTCTTCCTGCA	130	88.2
cassava4.1_010439	CGGAGAGAGGGCCTTGTTTA	ACCCAACTTCAAGTCAGGCA	162	84.8
cassava4.1_024813	GTGGCAAAGGGAGGTGATCT	GCCAAACTAGGGGATGCCATA	65	100.8
cassava4.1_003449	TGTTTGCTCTGGTTTGCTTGT	AGGGTCTTCAATAGCTGGCTT	95	109.4
cassava4.1_006445	GGACTGGACTTCGCAACATT	TTGCATCAATTGCACCATCT	147	94.8
cassava4.1_006884	AATGCGCTCCTACAACAAGC	GATCATCCGTAGCAGCCTCT	100	85.4
cassava4.1_006391	ATTATGCAAGCGGGACAAAC	ACTCCACCGTACATCCTTGC	64	102
cassava4.1_010236	GATGCCATTCATGCCTTTG	TCCGACCCTCGCTATCTTT	100	101.2
cassava4.1_012496	GCTTGTCATGGAAGGGTACAA	TTCCCACATCGGTAGCAATAG	86	96
cassava4.1_014646	AATGTGGCAAACAGGGTCTC	CTTGAAGGGTCTAGCGATGC	94	97.9
cassava4.1_003988	GACTGCATAAGAACGCGCTG	GATTTCTCTCCGGCCGACAA	127	94.5
cassava4.1_010665	AGTTGGAGGTGGAGGGTCTT	CATGGCTCGATCAACCTCTT	144	97.2
cassava4.1_014335	GTTTGATGAGCACCGGAAG	TCTTGGCCTGAGACTTGGA	62	100.5
cassava4.1_014799	GAGGTTGGAGTGGTGAGGAA	TCGCTCGCTTATAGCAGTCA	90	95.37
cassava4.1_006776	TGGTCAGCACATTTGTTCGT	AGCAGACCCCGTCATTGTAG	106	109.6
cassava4.1_017977	ATGGGTTGTTAGCGGCTCT	ATCTGCTCCGCCAACTTCT	114	110.1
cassava4.1_002641	CAGGGCATTGAGAAAAGTTCC	AGTTCTTTCATCCCCAGCAAA	80	95.1
cassava4.1_004051	GATTGACACACCACAGCCTG	CACAAGTGGTCTTCTGTGCC	68	83.3
cassava4.1_001927	GGTCCAAACGTGATGCAA	ATAGCCAATGCCAAGACCA	104	100

### Expression Stability of the Candidate Reference Genes

**FIGURE 1:**
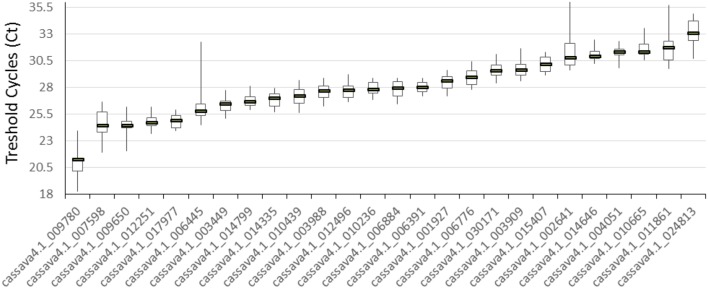
**Expression levels of 26 candidate reference genes in 21 different samples**. The median value and the minimum and maximum *C*_q_ of the 21 tissues or organs were calculated. Three technical replicates were used.

The 21 cDNA samples were analyzed through real-time RT-PCR by using the 26 primer pairs. The expression levels of the 26 candidate reference genes were determined as quantification cycles (*C*_q_ value; **Figure 1**). The genes displayed a relatively wide range of mean *C*_q_ values from 21 (*cssava4.1_009780*, *ACT7*) to 33.1 (*cassava4.1_024813*).

Two programs, namely, geNorm and NormFinder, were used to calculate the stability of expression of the candidate reference genes.

#### geNorm Analysis

**FIGURE 2:**
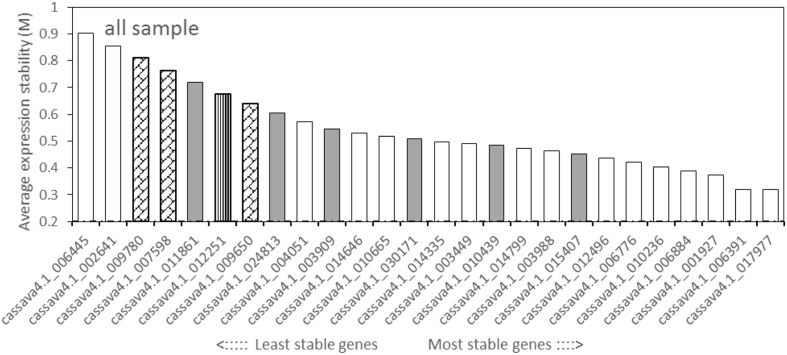
**Expression stability values (*M*) and ranking of the candidate reference genes for all tested samples based on geNorm algorithm**. The proposed cutoff *M* value was 1.5 because a low *M* value indicates a stable gene expression. The most stable genes are listed on the right and the least stable on the left (pin stripe, PP2A; oblique block, classic reference genes; gray filling, *Arabidopsis* references).

The geNorm tool calculates the gene expression stability value (*M*) and recommends on the basis of the *M* value below the threshold of 1.5. All of the tested candidate genes in this study displayed an *M* value less than 1.5 (**Figure 2**, Supplementary Figures S2 and S3), suggesting that the genes should be considered relatively stable. For all the tested samples, *cassava4.1_017977* and *cassava4.1_006391*, showed the lowest *M* value, but displayed the most stable profiles. *Cassava4.1_006884* was ranked fourth among the stable genes. *Cassava4.1_006445* and *cassava4.1_002641* are the least stable with the highest *M* value. *Cassava4.1_009780* (*ACT7*), *cassava4.1_007598* (*TUB*), *cassava4.1_009650* (*UBQ10*), and *cassava4.1_012251* (*PP2A*) displayed a relatively high *M* value and ranked last. The cassava orthologs of the six most stable *Arabidopsis* demonstrated varying performance (**Figure 2**).

For normally grown samples (Supplementary Figure S2), the four most stable reference genes ranked by geNorm were *cassava4.1_012496*, *cassava4.1_006391*, *cassava4.1_006884*, and *cassava4.1_017977*, whereas *ACT7*, *TUB*, *UBQ10*, and *PP2A* were the least stable genes. Among the drought stressed samples (Supplementary Figure S3), geNorm recommended *cassava4.1_014335* and *cassava4.1_006884* as the two most stable reference genes and *cassava4.1_006445* and *cassava4.1_002641* as the two least stable genes.

Pairwise variation *V*_2/3_ was less than 0.15 in all 21 normally grown samples, and drought-stressed samples (Supplementary Figures S4–S6), indicating that adding a second gene is necessary to obtain an accurate analysis, whereas addition of the third gene is optional.

#### NormFinder Analysis

**FIGURE 3:**
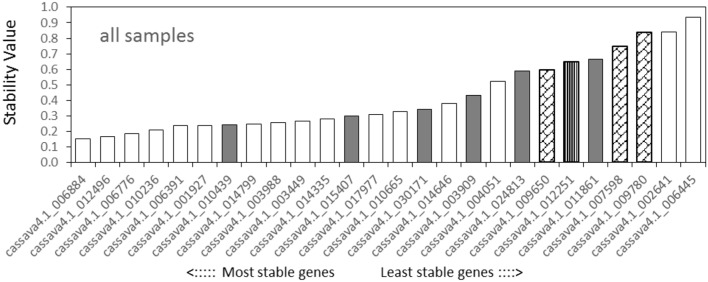
**Expression stability values and ranking of the candidate reference genes for all tested samples based on NormFinder algorithm**. The lowest stability value indicates the most stable expression within the examined gene set. The most stable genes are listed on the left and the least stable on the right (pin stripe, PP2A; oblique block, classic reference genes; gray filling, *Arabidopsis* references).

NormFinder analysis found that among the tested samples (**Figure 3**), cassava4.1_006884 was the most stable gene, followed by *cassava4.1_012496* and *cassava4.1_006776*, whereas the three least stable genes were *cassava4.1_006445*, *cassava4.1_002641*, and *cassava4.1_009780* (*ACT7*). For the drought stressed samples (Supplementary Figure S7), NormFinder suggested that *cassava4.1_00677*6 and *cassava4.1_003988* are the most stable genes. The NormFinder ranked *cassava4.1_006445* and *cassava4.1_002461* as the least stable genes in all tested samples and drought-stressed samples.

The NormFinder suggested that the four most stable genes among the normally grown samples were *cassava4.1_006776*, *cassava4.1_006391*, *cassava4.1_012496*, and *cassava4.1_006884*, whereas *cassava4.1_009780* (*ACT7*), *cassava4.1_007598* (*TUB*), *cassava4.1_009650* (*UBQ10*), and *cassava4.1_012251* (*PP2A*) were ranked as the least stable genes (Supplementary Figure S8).

#### Expression Profiling of Target Genes

In order to validate the effect of using different reference genes on normalizing the relative expression of other genes, *UGT85K4/UGT85K5* and *cassava4.1_004044* were used as samples. UGT85k4 and UGT85K5 are paralogs catalyzing *in planta* synthesis of cyanogenic glucosides (Kannangara et al., 2011). *UGT85K4* and *UGT85K5* share up to 96% sequence similarity and the same primer pair. Two cyanogenic glucosides, linamarin and lotaustralin, are distributed in all the parts of a cassava plant, except in seeds. *Cassava4.1_004044* is a homologous sequence of *Nitrate transporters NRT1*. Four reference genes or their combinations were used: *PP2A*, *ACT*, geometric average of the two genes indicated by geNorm in all samples (*cassava4.1_006391* and *cassava4.1_017977*), and *cassava4.1_06776* recommended by NormFinder in normal or drought stressed samples. *PP2A* is validated o be stably expressed in potyvirus-infected cassava samples, but less stably expressed in samples tested in this study. *ACT* is a traditional reference gene extensively used to normalize target genes. However, *ACT* was validated as an unstable reference gene in this study.

**FIGURE 4:**
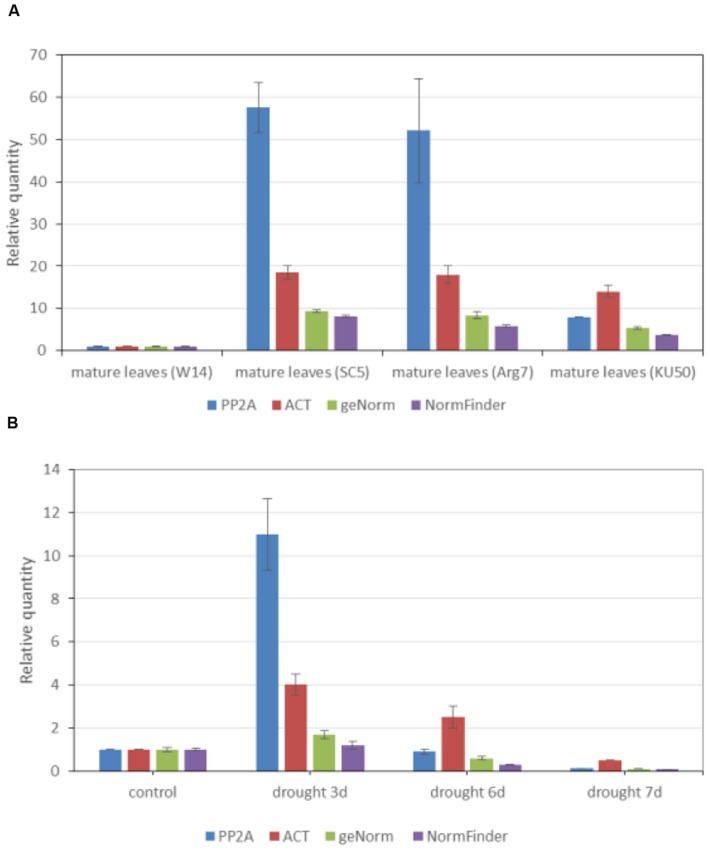
**Expression profiles of *UGT85K4/UGT85K5* in mature leaves of different varieties and of *cassava4.1_004044* in mature leaves of drought stressed plants**. Relative quantification of *UGT85K4/UGT85K5* in mature leaves of four varieties **(A)** and of *cassava4.1_004044* in mature leaves of drought stressed plants **(B)** by using the following genes as references: (i) PP2A, (ii) ACT, (iii) combinations of *cassava4.1_006391* and *cassava4.1_017977* as recommended by geNorm, and (v) *cassava4.1_006776* recommended by NormFinder. Results are expressed as a ratio of the value in mature leaves of W14 and mature leaves of normally watered plants, which is arbitrarily set to 1. Normalized values of *UGT85K4/UGT85K5* and *cassava4.1_004044* relative expression are given as average ± *SD*. *SD* refers to the standard deviation of three biological repeats.

Two data sets were used to calculate these target genes. Mature leaves of four normally grown plants were used to estimate the relative expression of *UGT85K4* and *UGT85K5* (**Figure 4A**). These plants are W14, SC5, Arg7, and KU50 varieties growing in field for 6 months. Drought-stressed samples were used to estimate the relative expression of *cassava4.1_004044* (**Figure 4B**). In greenhouse, the plants grown in 35 cm × 40 cm × 45 cm plots for 5 months were treated by withholding water for 3, 6, and 7 days. The control plants were watered with 2 L of water every day. The mature leaves of these plants were collected on the same day. Each repetitions was performed in triplicates.

Data on the relative expression of target genes normalized by relatively stable reference genes recommended by the two algorithms were consistent with the expression of both *UGT85K4/UGT85K5* (**Figure 4A**) and *cassava4.1_004044* (**Figure 4B**) in tissues. However, the result normalized by *PP2A* and *ACT* was the exception. W14 variety is a relatively wild variety, whereas SC5, Arg7, and KU50 are cultivar varieties (Wang et al., 2014). The mRNA of *UGT85K4/UGT85K5* was constitutively expressed in all varieties, with high expression in cultivars (**Figure 4A**). The use of *PP2A* as reference gene led to significant overestimation of the transcription level of *UGT85K4/UGT85K5* (**Figure 4A**) in mature leaves of SC5 and Arg7. In addition the use of *ACT* as reference gene led to an overestimation of *UGT85K4/UGT85K5* in mature leaves of SC5, Arg7, and KU50. In drought-stress experiments, mature leaves were collected from normally watered plant, and from plants drought stressed for 3, 6, and 7 days. Normalization using *PP2A* of *cassava4.1_004044* expression in drought-stressed samples led to a large overestimation of transcription level approximately 6.8–10 times higher than those obtained using the most stable genes as indicated by geNorm and NormFinder in drought-stressed samples after 3 days (**Figure 4B**). Moreover, the normalization procedure with *ACT* showed a relatively weak overestimation of *cassava4.1_004044* expression in mature leaves collected from plants drought stressed for 3, 6, and 7 days (**Figure 4B**). In this experiment, the novel stably expressed reference genes were used as control, and no remarkable changes in *cassava4.1_004044* transcription were observed under drought stress for 3 days. The use of *PP2A* and *ACT* as reference genes led to an overestimation of *cassava4.1_00404* under drought stress for 3 days. *PP2A* and *ACT* were poorly ranked by the two algorithms, independent of the experimental data set. Furthermore, overestimation of transcription level of *UGT85K4/UGT85K5* and *cassava4.1_004044* normalized by *PP2A* and *ACT* indicated the unstable expression of *PP2A* and *ACT* in various samples. These lines of evidence suggest that *PP2A* and *ACT* are unsuitable reference genes for gene expression studies in cassava. The use of gene and gene combination (best ranked by geNorm and NormFinder) as reference genes results in similar relative expression data.

## Discussion

### Selection of Candidate Reference Genes in Cassava

Real-time reverse transcription PCR (RT-PCR) has become an important method used for accurate expression profiling of selected genes (Nolan et al., 2006; VanGuilder et al., 2008). The use of suitable reference genes is a prerequisite to ensure reliable and relatively accurate data. Numerous studies have reported that traditional reference genes are not always stable in various plants, including *Arabidopsis* (Czechowski et al., 2005) and rice (Jain, 2009). Czechowski et al. (2005) showed that excellent reference genes can be identified from a large collection of comprehensive transcriptome data obtained from DNA-array hybridization studies; in addition, they have revealed some novel compatible reference genes, including those that encode a PP2A subunit, a coatomer subunit, and an ubiquitin-conjugating enzyme. Some studies selected some orthologs of the most stable reference gene in *Arabidopsis* validated by Czechowski et al. (2005) to be candidate target genes and they have obtained satisfactory results (Demidenko et al., 2011; Dekkers et al., 2012).

Sequence data are always used as important tools to select candidate reference genes. An EST database of chicory was used to obtain 18 potential reference genes (Delporte et al., 2015). Buckwheat floral transcriptome sequenced by 454 technology was used to obtain the sequences of candidate genes, indicating that the transcriptome sequence data provid a source of robust normalization genes (Demidenko et al., 2011). Cassava genome sequence was published on Phytozome^3^ (Prochnik et al., 2012). In addition, genome data of two other cassava cultivars were evaluated (Wang et al., 2014). Validating and obtaining a target gene sequence became easy because of the increasing number of genome and transcriptomic data are released. For example, the effective utilization of existing genome and transcriptome data facilitates research on tomato (Dekkers et al., 2012).

We did not only select six cassava orthologs of the most stable reference genes but also screened and chose 16 candidate genes by using the genome-wide identification method based on the transcriptome sequence data collected in our laboratory. Whether geNorm or NormFinder were used for analysis, some candidate genes that were selected from transcriptional data were ranked as the most stable genes. Neither traditional reference genes (such as *ACT*, *TUB*, *UBQ10*, and *PP2A*, which were selected in previous studies) nor cassava orthologs of most stable *Arabidopsis* genes were the best choices. We took advantage of the fact that large-scale transcriptomic data were available for many plant species, and our results demonstrated that these data were actually a useful pool of potential reference genes.

**FIGURE 5:**
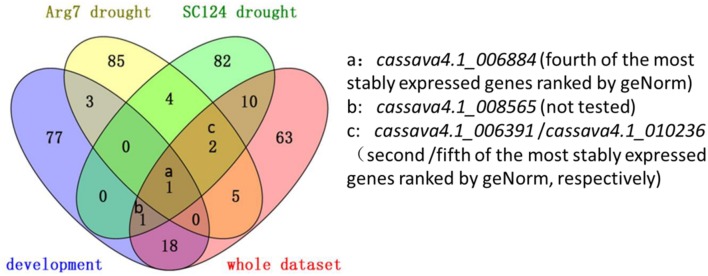
**Venn diagram of the statistically determined candidate reference genes**.

A total of 16 candidate genes were initially selected from the three series. After experimentation and comparison, the top 100 stably expressed genes were determined by using 32 transcriptomic datasets. Prediction from the three series and 32 whole datasets was partially coincided from a Venn diagram (**Figure 5**)^4^. For instance, *cassava4.1_006884* was ranked top 100 in the lists of the three series and the list of whole datasets (**Figure 5**). In addition this gene was suggested as one of the most stably expressed genes by two algorithms when tested in 21 samples (**Figures 2** and **3**) or in normally grown samples and drought-stressed samples (Supplementary Figures S2, S3, S7, and S8). Two invariant genes, namely, *cassava4.1_006391* and *cassava4.1_010236* were ranked out of top 100 in the developmental series, while ranked top 100 in two other drought stressed samples subsets and the whole dataset. However, two algorithms ranked *cassava4.1_006391* as top 2 or 5 in the normal developmental sample series and top 11 or 21 in the drought stress sample series. The two algorithms ranked *cassava4.1_010236* as the fifth or the fourth most stably expressed genes in all tested samples and top seven in drought-stressed samples. Those unstable candidates of the 16 selected genes tend to appear twice or once. Similar observation was noted in *Arabidopsis* (Czechowski et al., 2005). In *Arabidopsis*, Czechowski et al. (2005) divided more than 1000 Affymetrix ATH1 whole-genome GeneChip dataset into eight series. The top six stably expressed genes appear six or four times, and the genes of low rank tend to appear thrice or once. These results possibly indicate on the frequency of the candidate genes in the datasets and the stability of the tested samples. In addition, some genes do not demonstrate such a relationship. For instance, *cassava4.1_001927* was ranked top 100 in the developmental series but not in other series. Two algorithms ranked this gene as top 4 or 5 in the normal developmental sample series but as top 14 or 15 in the drought-stressed sample series. These unclear relationship between computational prediction and experimental evaluation makes selection of candidate stably expressed genes difficult. One way to narrow down the search is to first focus on the high-frequency genes in the top 100 list. This strategy would likely reduce work and uncertainty.

### Comparison of Candidate Reference Genes in Cassava and the Limitation

A total of 21 cassava samples were used to investigate the stability of expression of the 26 candidate reference genes selected from previous reports or from transcriptome data. These 21 samples contained different tissues and organs from normally grown plants, diseased plants, or drought-stressed plants. The experimental data were divided into three series: total samples, normally grown samples, and drought-stressed samples. Similar to the result in *Arabidopsis*, all of the best reference genes for cassava evaluated in this study were selected from 32 transcriptome data. In addition, the result for normally grown samples is similar to that of all other samples. geNorm ranked three pair combinations for three different series. After investigating the first four stably expressed genes in the three experimental set, *cassava4.1_017977* and *cassava4.1_006884* are the relatively best choice because of their high frequency in the top four stable genes in three experimental set as revealed by geNorm. NormFinder ranked *cassava4.1_006884*, *cassava4.1_012496*, and *cassava4.1_006776* after investigating all the 21 tested samples and *cassava4.1_006776* after analyzing the drought stress and normally grown samples. The result of normalization of *UGT85K4/UGT85K5* and *cassava4.1_004044* has proven that the genes indicated by the two algorithms are better than *ACT* (traditional reference gene) and *PP2A* (stable reference gene for quantitating potyvirus in cassava). For example, the use of the novel reference genes as control showed that expression of *cassava4.1_004044* under drought stress for 3 days was unremarkably changed. However, normalization by *PP2A* and *ACT* will lead to overestimation of *cassava4.1_004044* under a 3-day drought stress. This result corresponded to those of the two algorithms.

To choose the suitable reference genes, we suggest that the transcript level of target genes and candidate reference genes must be evaluated. One valid method is to compare their quantification (*C*_q_) values. Degree of gene expression varies and is reflected by different *C*_q_ values in a specific concentration of cDNA pool. For instance, *ACT* always displays a higher expression than most of other genes exhibiting a lower *C*_q_ value. In addition, *18S ribosomal RNA* and *glyceraldehyde-3-phosphate dehydrogenase* also show a relatively high transcription level. By contrast, most transcription factors are rarely expressed and show lower *C*_q_ values than most other genes. The use of a highly expressed gene to normalize a rarely expressed gene and vice versa will produce errors. Comparison of the *C*_q_ values of a target gene and a highly expressed gene and some of these potential reference genes in one experiment will allow one to make a quick judgment of their mRNA transcript levels. We then can choose suitable reference genes showing a similar *C*_q_ value to normalize the expression of target genes.

However, the use of the validated reference genes is limited. First, those lists were based on 32 dataset only and not on more than 100 dataset or even 1000 dataset. The accuracy of the statistical prediction increases in direct ratio to amount of datasets. When new transcriptomic data were calculated, the lists will change, and the new prediction will become more accurate. Second, these reference genes were validated in specific varieties and conditions. The specific result should be investigated for new promotion. In addition, different varieties and experimental conditions may influence gene expression.

### Strategy to Select Candidate Reference Genes

The next-generation sequencing technology has been rapidly developed to analyze the transcriptome database. Compared with the EST database, the transcriptome database is advantageous in terms of reflecting the relative gene expression level. Sufficient and various transcriptome data produce a competent platform to select candidate reference genes (Czechowski et al., 2005). In *Arabidopsis*, mass data from more than 1000 GeneChip studies were used to screen potential reference genes (Czechowski et al., 2005). Unfortunately, to date, only a handful of transcriptome data have been revealed in major plant species. The less data we have, the lower the effectiveness of the data will be, and the less novel candidates we can obtain. For instance, when only four transcriptome data were used in *Oxytropis ochrocephala* Bunge, 9 of the 12 candidates were orthologs of traditional reference genes: *18S ribosomal RNA*, *ACT*, *GAPDH*, and *TUB* (Zhuang et al., 2015). However, when the transcriptome data of 10 *Brassica napus* L. tissues were used, 13 novel candidates were found and most of them demonstrated a more stable expression than the traditional reference genes *ACT7* and *GAPDH* (Yang et al., 2014). This study has taken advantage of three experimental sets consisting of 32 experimental data and reasonably showed that an experimental setup containing more than 10 data is useful to seek parallel that ranked genes appeared in the list of top 100 stable genes. Thus, selecting potential reference genes by using more than 10 transcriptome data for a species is reasonable.

Moreover, many studies on new research objectives are based on no or little transcriptome information. A preliminary and temporary suggestion is to consider orthologs of the highly expressed traditional reference genes and the orthologs of the most stable genes in *Arabidopsis* (Czechowski et al., 2005). This strategy has been successfully and widely used in new research objects: *Cichorium intybus* (Delporte et al., 2015), *Buglossoides arvensis* (Gadkar and Filion, 2015), *Setaria viridis* (Lambret-Frotte et al., 2015), *Actinidia deliciosa* (Petriccione et al., 2015), and *Lolium multiflorum* (Annual Ryegrass; Wang et al., 2015). The orthologs of some traditional genes have been validated in those plants and some of which demonstrate a relatively stable expression. Traditional reference genes contain abundant mRNA copies and are easy to be cloned by degenerate primers. Hence, they are potential options for new research objects.

The evaluated reference genes are stably expressed in the tested varieties and conditions. Before using these results, selecting some candidates to test their stability in each specific experimental conditions is advisable. This strategy will increase the precision of evaluation.

## Conclusion

This analysis revealed that hundreds of genes in the cassava genome are more stably expressed at low levels than traditional reference genes under specific varieties and conditions. Furthermore, transcriptional sequence data are suitable sources for selecting candidate control genes.

## Author Contributions

MH and WW conceived, designed, and performed the experiments. MH and WH wrote the paper. Meanwhile, MH analyzed the data. ZX and XZ contributed to the transcriptome data.

## Conflict of Interest Statement

The authors declare that the research was conducted in the absence of any commercial or financial relationships that could be construed as a potential conflict of interest.

## Footnotes

1 http://phytozome.jgi.doe.gov/pz/portal.html

2 http://primer3.ut.ee/

3 http://phytozome.jgi.doe.gov/pz/portal.html

4 http://bioinfogp.cnb.csic.es/tools/venny/index.html
