# Supplementary material for: Validation of Reference Genes for Relative Quantitative Gene Expression Studies in Cassava (Manihot esculenta Crantz) by Using Quantitative Real-Time PCR
Source: Front Plant Sci. 2016 May 19;7:680. doi: 10.3389/fpls.2016.00680 (PMC4871855; doi:10.3389/fpls.2016.00680)
Supplement: Supplementary file 2 [file Data_Sheet_2.DOCX]

***Supplementary Material***

**Validation of Reference Genes for Relative Quantitative Gene Expression Studies in Cassava using Quantitative Real-Time PCR**

**Meizhen Hu^1,2,3^, Wenbin Hu^4^, Zhiqiang xia^2^, Xincheng Zhou^2^, Wenquan Wang^1,2*^**

^1^College of Agriculture, Hainan University, Haikou, China

^2^The Institute of Tropical Biosciences and Biotechnology, Chinese Academy of Tropical Agriculture Sciences, Haikou, China

^3^Key Laboratory of Biology and Genetic Resources of Tropical Crops, Ministry of Agriculture, Haikou, China

^4^Tropical Crops Genetic Resources Institute, Chinese Academy of Tropical Agriculture Sciences, Danzhou, China

*** Correspondence:** Wenquan Wang, College of Agriculture, Hainan University, Haikou, China.

wangwenquan@itbb.org.cn

**Supplemental Figure 1. Dissociation Curves for PCR products amplified with the Primer Pairs shown in Table 2.**


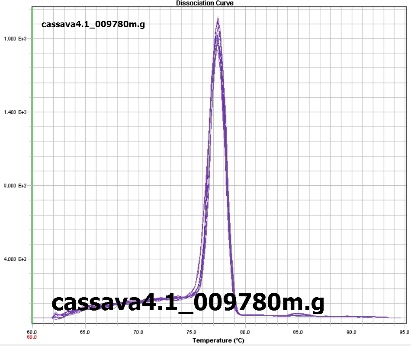

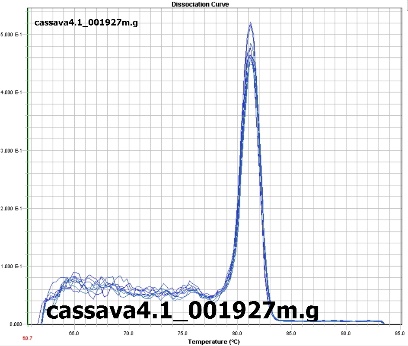

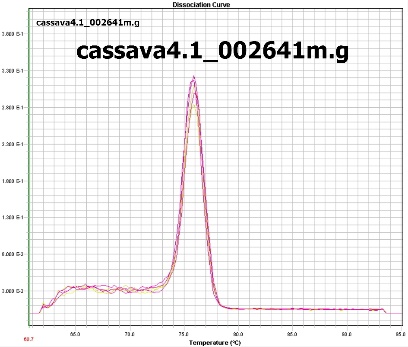

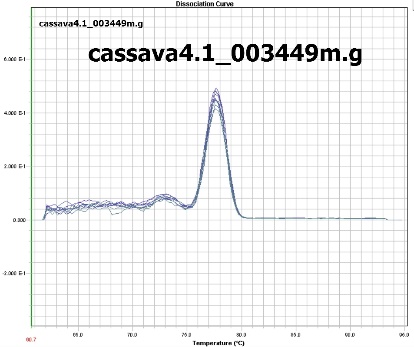

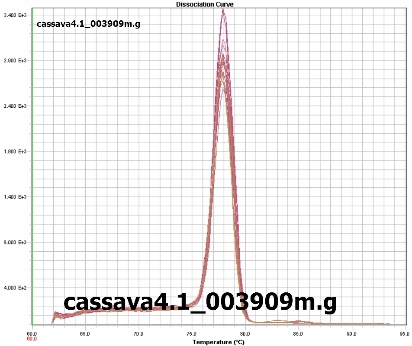

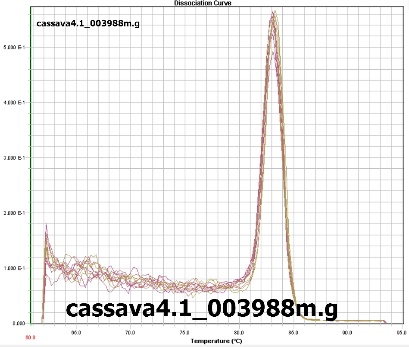


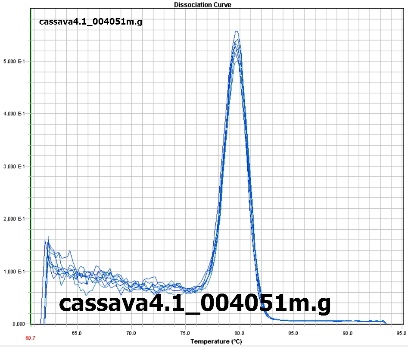

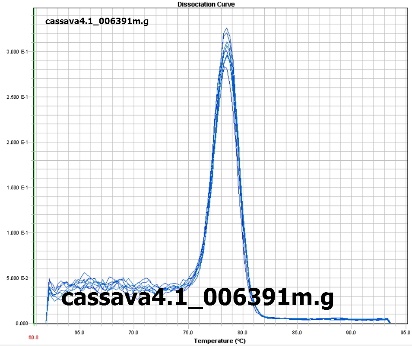

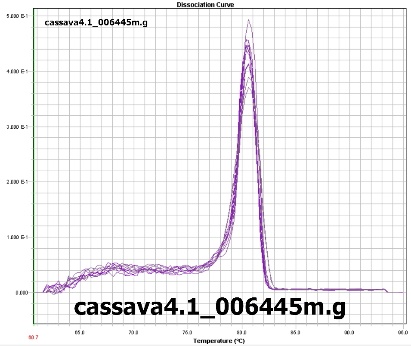

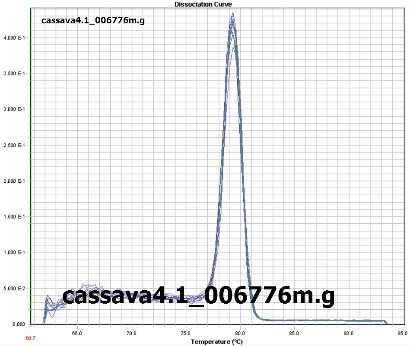

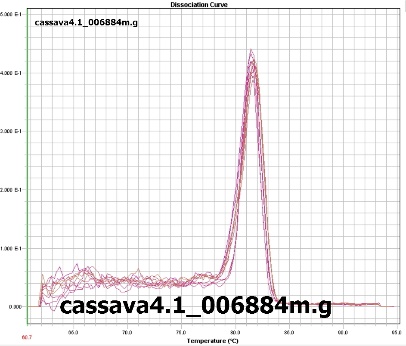

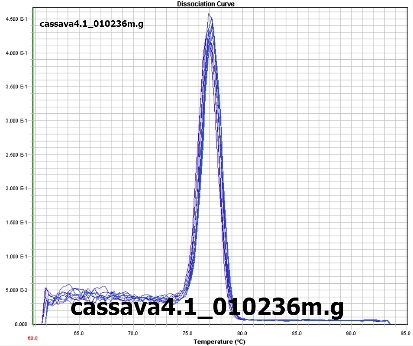

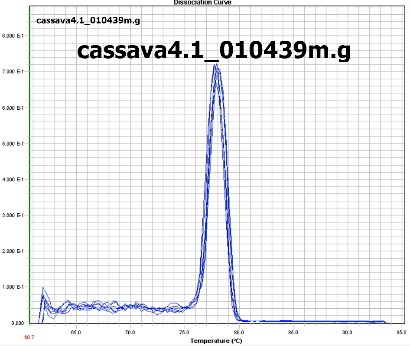

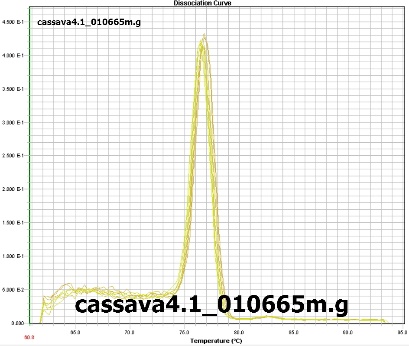

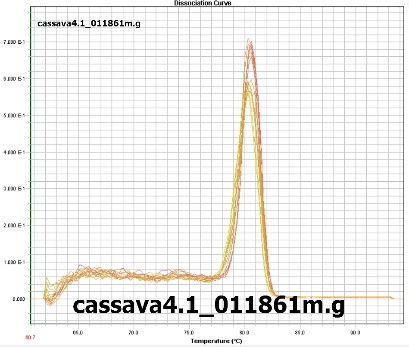

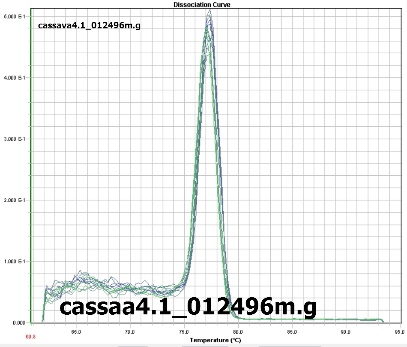

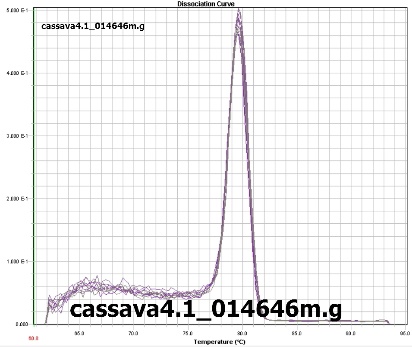

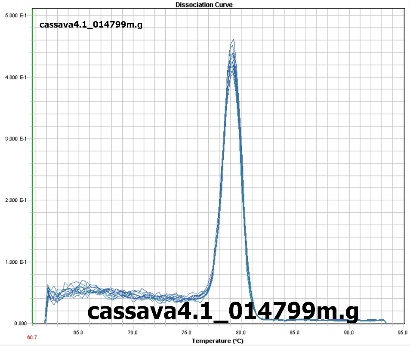

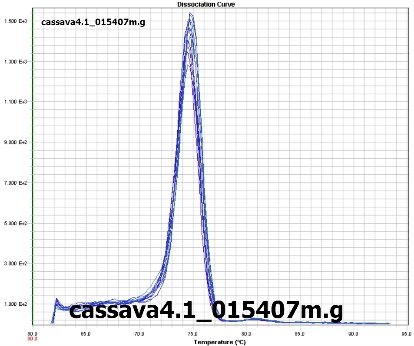

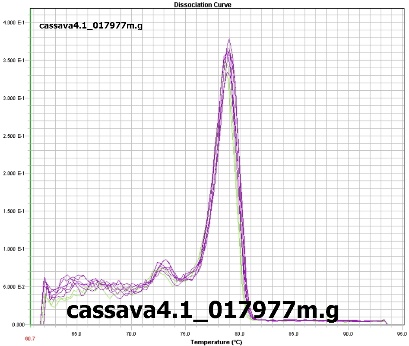

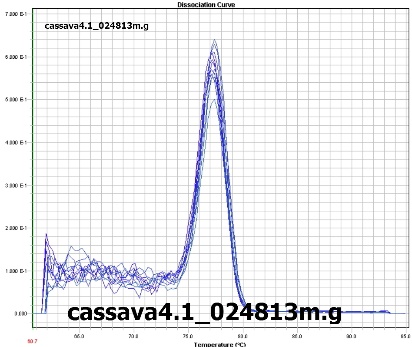

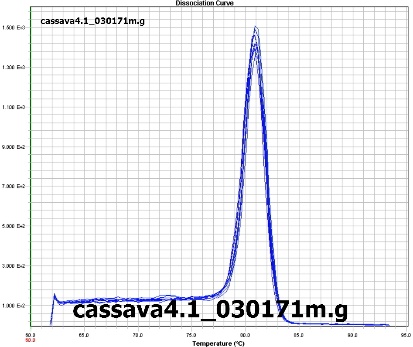

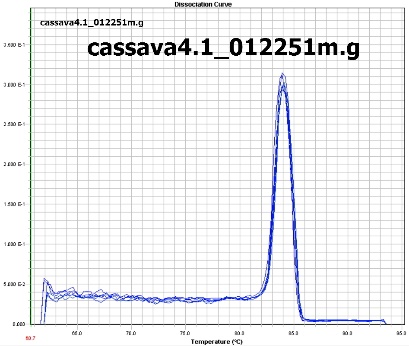

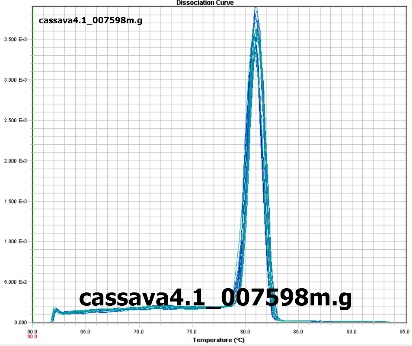

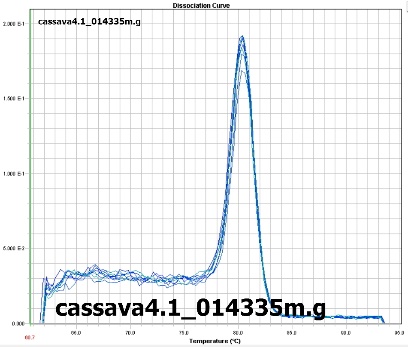

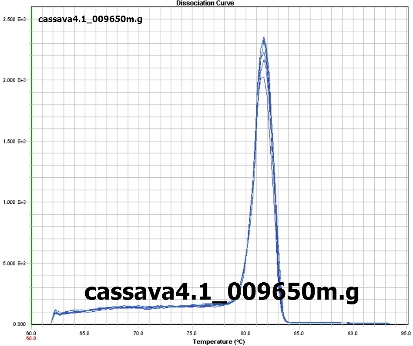


**Supplemental Figure 2 | Expression stability values (M) and ranking of the candidate reference genes for normal grown samples based on geNorm algorithm.**

The cutoff M value was proposed to be 1.5, a lower M value indicated more stable gene expression. The most genes are listed on the right and least stable on the left. (pin stripe, PP2A; oblique block, classic reference genes; gray filling, Arabidopsis references)

**Supplemental Figure 3 | Expression stability values (M) and ranking of the candidate reference genes for drought stressed samples based on geNorm algorithm.**

The cutoff M value was proposed to be 1.5, a lower M value indicated more stable gene expression. The most genes are listed on the right and least stable on the left. (pin stripe, PP2A; oblique block, classic reference genes; gray filling, Arabidopsis references)

**Supplemental Figure 4 | Pairwise variation (V_n_/V_n+1_) analysis of the candidate reference genes for all samples.**

Each variation was below 0.15, that indicated an additional reference genes is not necessary.

**
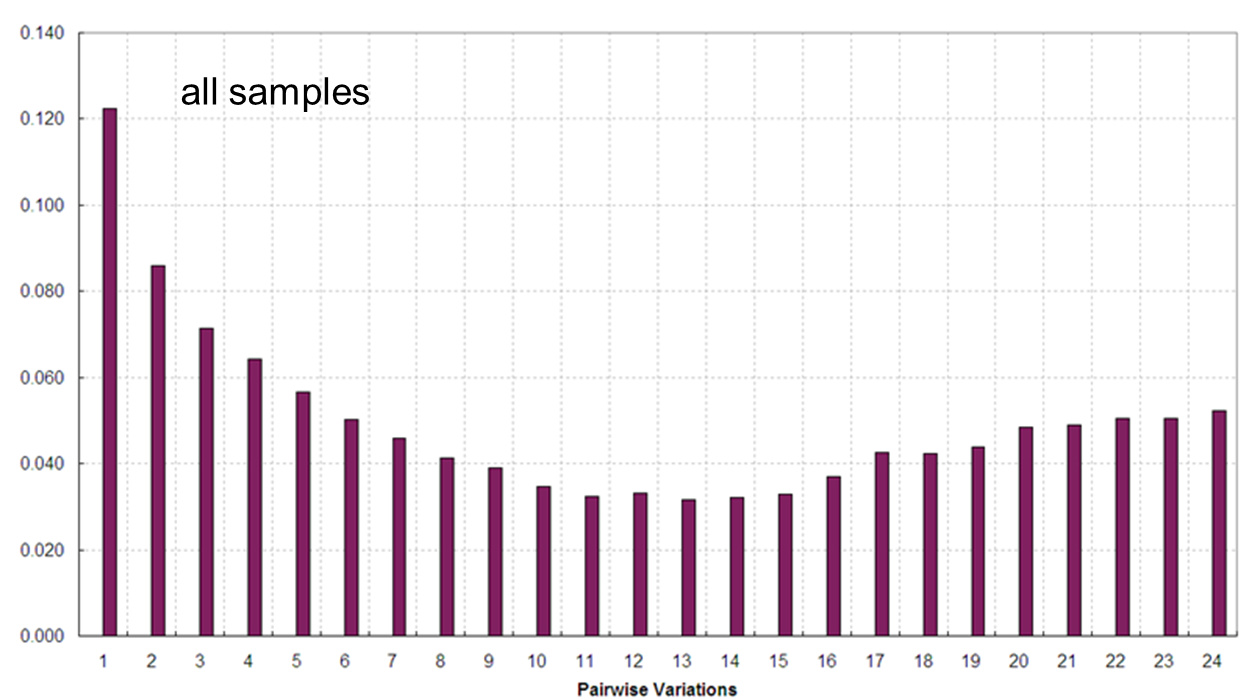
**

**Supplemental Figure 5 | Pairwise variation (V_n_/V_n+1_) analysis of the candidate reference genes for normal grown samples.**

Each variation was below 0.15, that indicated an additional reference genes is not necessary.

**
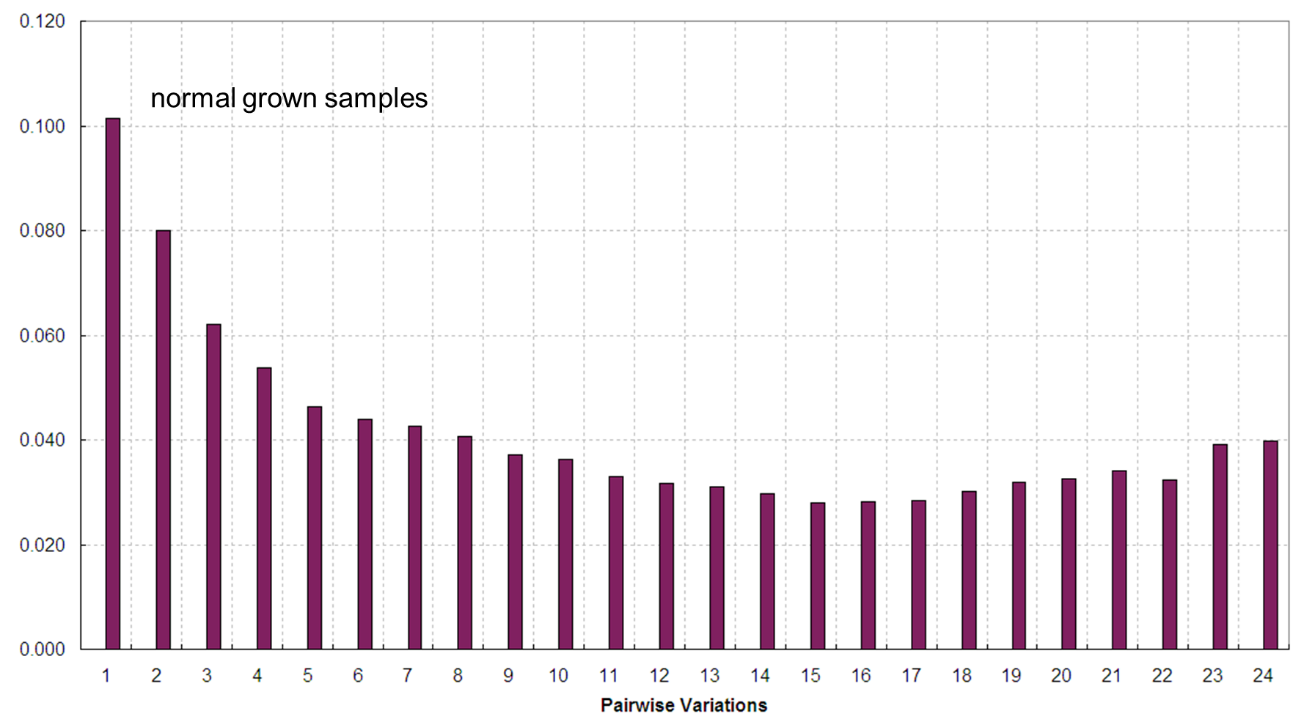
**

**Supplemental Figure 6 | Pairwise variation (V_n_/V_n+1_) analysis of the candidate reference genes for drought stressed samples.**

Each variation was below 0.15, that indicated an additional reference genes is not necessary.

**
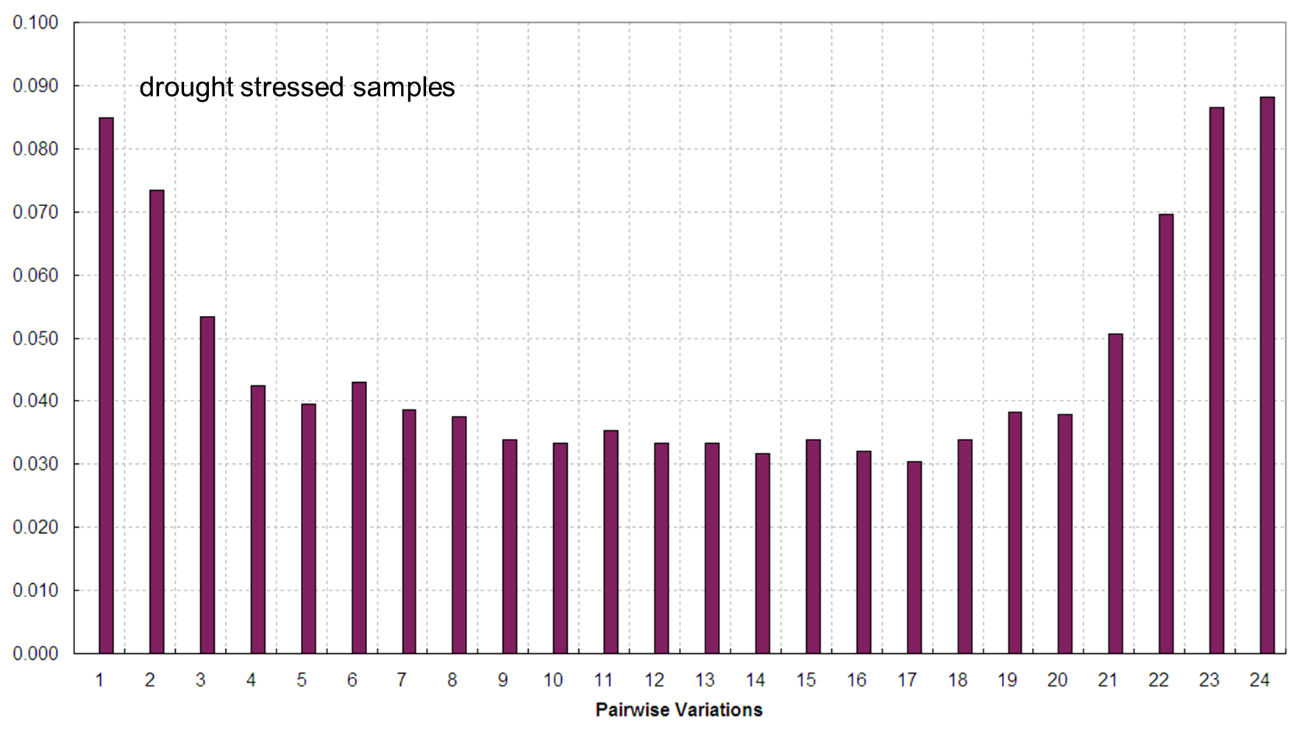
**

**Supplemental Figure 7 | Expression stability values and ranking of the candidate reference genes for drought stress samples based on Normfinder algorithm.**

The lowest stability value indicates the most stable expression within the gene set examined. The most stable genes are listed on the left and the least stable on the right. (pin stripe, PP2A; oblique block, classic reference genes; gray filling, Arabidopsis references)

**Supplemental Figure 8 | Expression stability values and ranking of the candidate reference genes for normal developmental samples based on NormFinder algorithm.**

The lowest stability value indicates the most stable expression within the gene set examined. The most stable genes are listed on the left and the least stable on the right. (pin stripe, PP2A; oblique block, classic reference genes; gray filling, Arabidopsis references)

**Supplemental Table 1. Profiles of cassava transcriptome data used to screen 16 candidate housekeeping genes. (Tab marks transcriptome data of samples in supplemental Excel 1. Expression level is defined by fragments Per Kilobase of exon model per Million fragments mapped, FPKM)**

Panel Descriptions:

1. Developmental series
2. Drought stress for Arg7
3. Drought stress for SC124

| 1. Developmental series | | |
| --- | --- | --- |
| **Cassava variety and tissue** | **tab** | **treatment** |
| Arg7 tuberous | Arg7_ETR | 90 days planted in fields |
| Arg7 tuberous | Arg7_MTR | 150 days planted in fields |
| Arg7 tuberous | Arg7_LTR | 240 days planted in fields |
| Arg7 leaf | Arg7_FL | 150 days planted in fields |
| KU50 tuberous | KU50_ETR | 90 days planted in fields |
| KU50 tuberous | KU50_MTR | 150 days planted in fields |
| KU50 tuberous | KU50_LTR | 240 days planted in fields |
| KU50 leaf | KU50_FL | 150 days planted in fields |
| W14 tuberous | W14_MTR | 150 days planted in fields |
| W14 leaf | W14_FL | 150 days planted in fields |

| 1. Drought stress for Arg7 | | |
| --- | --- | --- |
| **Cassava variety and tissue** | **tab** | **treatment** |
| Arg7 stem cortex | Arg7_DSCC3Dck | 183 days after cutting in a plot with 35 × 40 × 45 (normal watered) |
| Arg7 stem cortex | Arg7_US3D | 183 days after cutting in a plot with 35 × 40 × 45 (drought treated for 3 days) |
| Arg7 young stem | Arg7_US12Dck | 192 days after cutting in a plot with 35 × 40 × 45 (normal watered) |
| Arg7 young stem | Arg7_US12D | 192 days after cutting in a plot with 35 × 40 × 45 (drought treated for 12 days) |
| Arg7 stem cortex | Arg7_DSCC12Dck | 192 days after cutting in a plot with 35 × 40 × 45 (normal watered) |
| Arg7 stem cortex | Arg7_DSCC12D | 192 days after cutting in a plot with 35 × 40 × 45 (drought treated for 12 days) |
| Arg7 young stem | Arg7_US18Dck | 198 days after cutting in a plot with 35 × 40 × 45 (normal watered) |
| Arg7 stem cortex | Arg7_US18D | 198 days after cutting in a plot with 35 × 40 × 45 (drought treated for 18 days) |
| Arg7 stem xylem | Arg7_DSCC18Dck | 198 days after cutting in a plot with 35 × 40 × 45 (normal watered) |
| Arg7 stem xylem | s Arg7_DSCC18D | 198 days after cutting in a plot with 35 × 40 × 45 (drought treated for 18 days) |

| 1. Drought stress for SC124 | | |
| --- | --- | --- |
| **Cassava variety and tissue** | **tab** | **treatment** |
| SC124 functional leaf | SC124-1 | 181 days after cutting in a plot with 35 × 40 × 45 (drought treated for 1 days) |
| SC124 functional leaf | SC124-20 | 200 days after cutting in a plot with 35 × 40 × 45 (drought treated for 20 days) |
| SC124 young stem | SC124-US3D | 183 days after cutting in a plot with 35 × 40 × 45 (drought treated for 3 days) |
| SC124 stem cortex | SC124-DSC3Dck | 183 days after cutting in a plot with 35 × 40 × 45 (normal watered) |
| SC124 stem xylem | SC124-DSCC3Dck | 183 days after cutting in a plot with 35 × 40 × 45 (normal watered) |
| SC124 stem xylem | SC124-DSCC3D | 183 days after cutting in a plot with 35 × 40 × 45 (drought treated for 3 days) |
| SC124 young stem | SC124-US12Dck | 192 days after cutting in a plot with 35 × 40 × 45 (normal watered) |
| SC124 young stem | SC124-US12D | 192 days after cutting in a plot with 35 × 40 × 45 (drought treated for 12 days) |
| SC124 stem cortex | SC124-DSC12D | 192 days after cutting in a plot with 35 × 40 × 45 (drought treated for 12 days) |
| SC124 young stem | SC124-US18D | 198 days after cutting in a plot with 35 × 40 × 45 (drought treated for 18 days) |
| SC124 stem cortex | SC124-DSC18Dck | 198 days after cutting in a plot with 35 × 40 × 45 (normal watered) |
| SC124 stem xylem | SC124-DSCC18D | 198 days after cutting in a plot with 35 × 40 × 45 (drought treated for 18 days) |

**Supplemental Table 2. Profiles of 21 tested samples used to evaluate candidate reference genes.**

Panel Descriptions:

1. Normally grown samples
2. Drought stress samples
3. Samples that suffered from disease or nitrogen deficiency

(D) Normally grown samples

| cassava variety | tissue | development stage |
| --- | --- | --- |
| KU50 | young leaves | 10 weeks |
| KU50 | fibrous roots | 10 weeks |
| KU50 | tuberous roots | 4 months |
| KU50 | mature leaves | 6 months |
| KU50 | petiole | 6 months |
| KU50 | mature leaves | 8 months |
| KU50 | tuberous roots | 8 months |
| KU50 | tuberous roots | 9.5 months |
| Rongyong9 | tuberous roots | 3 months |
| SC124 | tuberous roots | 6 months |
| SC5 | tuberous roots | 6 months |
| Arg7 | flowers |  |

(E) Drought stress samples

| cassava variety | tissue | treatment |
| --- | --- | --- |
| KU50 | all fully expanded leaves | aquaponics with 0.5 × Afdaling nutrient solution for 6 weeks, and then treated with 10% PEG6000 for 24 h |
| W14 | mature leaves | grown in 35 × 40 × 45 cm plots for 5 months, then withholding water treatment for 3 days |
| Arg7 | mature leaves | grown in 35 × 40 × 45 cm plots for 5 months, then withholding water treatment for 3 days |
| KU50 | mature leaves | grown in 35 × 40 × 45 cm plots for 5 months, then withholding water treatment for 6 days |
| Arg7 | mature leaves | grown in 35 × 40 × 45 cm plots for 5 months, then withholding water treatment for 7 days |
| SC124 | stems | grown in 35 × 40 × 45 cm plots for 5 months, then withholding water treatment for 4 days |

1. Samples that suffered from disease or nitrogen deficiency

| cassava variety | tissue | treatment |
| --- | --- | --- |
| KU50 (6 months) | leaves | infected with *cassava brown leaf spot* |
| KU50 (3 months) | leaves | infected with *Mononchellus mcgregori* |
| KU50 | leaves | aquaponics with 0.5 × Afdaling nutrient solution for 6 weeks, then aquaponics with the solution but without NH_4_NO_3_ for 2 weeks |
